# Editorial: Biochemical Reactions in Cytomimetic Media

**DOI:** 10.3389/fmolb.2019.00145

**Published:** 2020-01-09

**Authors:** Germán Rivas, Allen P. Minton

**Affiliations:** ^1^Centro de Investigaciones Biológicas, CSIC, Madrid, Spain; ^2^Laboratory of Biochemistry and Genetics, NIDDK, NIH, Bethesda, MD, United States

**Keywords:** cellular biochemistry, intracellular organization, molecular interactions, macromolecular crowding, liquid-liquid phase separation

Biochemical reactions have historically been studied in purified solutions that that are dilute in macromolecules and contained in large vessels. During the last several decades it has become increasingly clear that physiological media differ substantially from the solutions in which such studies are carried out. The fluid component of cells is distributed among numerous microenvironments that are highly heterogeneous in both composition and spatial organization. These factors have been shown to significantly influence the rates and equilibria governing many biochemical reactions. The results of recent efforts to characterize the behavior of individual proteins and other macromolecules within living cells are difficult to interpret unambiguously due to the complexity of the system. The study of biochemical kinetics and equilibria in well-characterized media designed to incorporate one or more (but not all) elements of the complexity of living cells, termed cytomimetic media, has been advocated as a means for bridging the gap between traditional *in vitro* studies and the more recent attempts to study these reactions in intact cells. This Special Issue of Frontiers in Biomolecular Sciences contains several articles in which different elements of environmental complexity are explored and discussed from theoretical and/or experimental points of view.

One of the elements of complexity is the presence of locally high concentrations of multiple species of macromolecules, which may interact with each other both specifically and non-specifically. Such interactions affect both the state of association and the chemical potential, or reactivity, of individual macromolecular species. Nguemaha et al. have utilized atomically detailed computer simulations to estimate the concentration-dependent free energy of transferring each of eight dilute “test” proteins from dilute solution to concentrated solutions of one of two concentrated “crowder” proteins. The simulation allows decomposition of the total transfer free energy into contributions from “hard” or steric repulsive interactions and “soft” or longer-ranged electrostatic or solvent-mediated interactions. Hoppe and Minton utilize an approximate analytical solution for the concentration-dependent transfer free energy of macromolecules interacting via a model square well-potential in order to calculate the effect of non-specific interactions on the concentration dependence of light scattering, sedimentation equilibrium, osmotic pressure, and liquid-liquid phase transitions of concentrated protein solutions. Their results show that this highly simplified interaction potential can recapitulate many experimentally observed phenomena as well as some key results of the atomistic simulations.

A second element of intracellular complexity is the presence of multiple microenvironments in which reactions may take place. Membraneless organelles, representing one class of these microenvironments, are thought to arise through liquid-liquid phase transitions. Nakashima et al. review theoretical models for the formation of incompatible phases and factors affecting their compositions, including salt concentration, pH, temperature, and the strength of intermolecular interactions. They further discuss factors regulating the partitioning of biomolecular reactants between the two phases, and the consequences of partitioning for reaction rates and equilibria in each of the phases.

A third element of complexity in the cellular environment is the dynamic nature of this environment, which is subject to changes with experimental conditions. Gnutt et al. review the effects of induced differentiation of neuronal cells and induced proteostasis of HeLa cells on the thermal stabilities of a synthetic folding sensor and a labeled mutant of superoxide dismutase. They report that these changes result in destabilization of the labeled sensor species. Because the analysis of the data is based upon signals averaged over the whole cell, the authors recommend development of cytomimetic media for a more detailed analysis.

A fourth element of complexity in the cellular interior is the presence of mobile and stationary obstacles to the free diffusion of macromolecular reactants. Schavemaker et al. review a number of factors affecting the rate of diffusional transport of macromolecules, which include transient binding to other species as well as the necessity of tortuous trajectories to bypass obstacles. The authors present examples of diffusion-limited reactions in small prokaryotic cells and point out that the functioning of certain intracellular oscillatory dynamic systems depends crucially upon the maintenance of diffusional rates.

## Author Contributions

GR and AM wrote the manuscript.

### Conflict of Interest

The authors declare that the research was conducted in the absence of any commercial or financial relationships that could be construed as a potential conflict of interest.

